# Handling Post-COVID-19 Sequelae: A Need for Multispecialty Approach

**DOI:** 10.3389/fpubh.2022.843329

**Published:** 2022-02-09

**Authors:** George Makrydakis, Lamprini Markaki, Emmanouil-Dimitrios Manikas, Ioannis Ntalas, Nikolaos Spernovasilis

**Affiliations:** ^1^Private Neurology Practice, Athens, Greece; ^2^First Department of Pediatrics, “Agia Sophia” Children's Hospital, School of Medicine, National and Kapodistrian University of Athens, Athens, Greece; ^3^Private Endocrinology Practice, Heraklion, Greece; ^4^London Cardiac CT Academy, London, United Kingdom; ^5^School of Medicine, University of Crete, Heraklion, Greece; ^6^German Oncology Center, Limassol, Cyprus

**Keywords:** post-COVID-19, sequelae, infectious disease specialist, multispecialty management, primary care, pandemic

One of the most worrying aspects of coronavirus disease 2019 (COVID-19) pandemic are the post-COVID-19 sequelae, affecting a significant proportion of both asymptomatic and symptomatic individuals with severe acute respiratory coronavirus virus 2 (SARS-COV-2) infection. These sequelae include a wide range of new, ongoing, recurring multisystemic health problems that were developed during or after initial viral infection. The global health body is deeply concerned not only by the still unknown burden of the overall post-COVID-19 sequelae, but also by the tremendous gaps in medical knowledge regarding many of these conditions and the healthcare and welfare resource allocation (HWRA) required for their management. Science should move with dispatch in addressing this problem in a multidisciplinary manner, otherwise the aforementioned gaps may quickly be filled by quackery and pseudoscience (1).

Until further research provides clinically significant diagnostic and therapeutic implements to approach post-COVID-19 sequelae, continuously integrating multispecialty consultancy is of paramount importance for patient care and welfare evaluation toward rehabilitation and societal reintegration. Since many of these conditions are similar to long-term health problems observed after SARS or Middle East respiratory syndrome (MERS) (2), clinical management paths may have been already drawn. Also, the fact that relevant experience on handling infectious and post-infectious sequelae exist from tuberculosis and HIV epidemics (3, 4), infectious disease specialists should be at the core of this multidisciplinary collaboration, at least during the initial stages of the post-acute period after COVID-19. In most countries of the world, infectious disease medicine is a subspecialty of pediatrics and internal medicine, allowing infectious disease specialists to act as ideal multispecialty coordinators in diagnostics, care and welfare guidance of children, adolescents and adults with post-COVID-19 sequelae.

Focusing on infectious disease expertise for post-COVID-19 sequelae management is a scientifically sound solution. Without effective and readily available outpatient treatments for many of these sequelae, entangling primary care providers into roles of care not yet matured may further deplete resources from diseases and disorders that primary care is already suited to handle, thus causing a generalized instability in healthcare systems. Further support to infectious disease expert leading of the multispecialty evaluation during the initial stages of post-COVID-19 sequelae is emphasized by examining the post-COVID-19 impairment repertoire in neurological, cardiorespiratory, endocrine and further aspects involving both pediatrics and internal medicine.

Aside from the well-known anosmia and parosmia, complex COVID-19 neurological impairment involving central (e.g., central demyelination, seizures, encephalopathy/encephalitis, neurocognitive dysfunction, strokes), peripheral (e.g., Guillain-Barré syndrome/other neuropathies, neuralgias, myopathy, myositis), and autonomic (e.g., dysautonomia, temperature and exercise intolerance) nervous systems requiring specialist involvement has been radiologically, functionally and neuropathologically verified, further increasing long-term post-infectious disability (5, 6). Notably, long-term neurological aftermath of COVID-19 is still an unexplored territory, a true challenge regarding its rehabilitation and an asymmetrical risk to HWRA. That, without including the all-important cost of related mental disorders and overall wellbeing (7, 8).

A considerable proportion of hospitalized COVID-19 patients will experience long-term pulmonary post-discharge sequelae, which may include impaired pulmonary diffusion capacities and abnormalities on imaging suggestive of pulmonary fibrosis (9), thus requiring a frequent follow-up by chest physicians. Regarding the cardiovascular system, in many patients post-COVID-19 myocardial injury can be suspected by persistent symptoms like dyspnea, easy fatigue on effort, muscle weakness and chest pain combined with abnormal troponin. Fulminant myocarditis has been reported in COVID-19 patients after weeks of undetectable viral load and symptoms amelioration, while some patients diagnosed with disease of mild severity have died due to cardiac arrest (10). Cardiomyopathy provoked by systemic hyperinflammation, coronary thrombotic and plaque rupture events, microvascular injury due to disseminated intravascular coagulation and thrombosis, supply–demand mismatch or hypoxia, and direct viral cardiotoxicity can be some of the mechanisms underlying troponin elevation in post-COVID-19 patients (11). Whenever a myocardial injury is discovered in post-COVID-19 patients, it should be further investigated with electrocardiogram, transthoracic echocardiogram (TTE), and cardiovascular magnetic resonance (CMR) scan. A close follow-up with TTE and a repeated CMR scan is usually indicated within 3–6 months post-discharge in patients with persistent elevated troponin, even if the first CMR scan does not show inflammation, myocardial fibrosis or scar.

With regard to the endocrine system, the pandemic has affected the way most endocrinological diseases are treated. Current guidance statements are based rather on expert consensus and are tailored to individual circumstances and local expertise. Patients with endocrinological conditions (diabetes mellitus, obesity, undernourishment, adrenal insufficiency, congenital adrenal hyperplasia, pituitary insufficiency) present increased vulnerability to SARS-CoV-2 infection, severe infection and/or death (12). Furthermore, SARS-CoV-2 has been implicated through several mechanisms (direct viral injury, immunological and inflammatory damage) in various new-onset endocrine diseases: development of type 1 diabetes mellitus, worsening of glycemic control in pre-existing type 2 diabetes mellitus, primary Leydig cell damage, critical illness-related corticosteroid insufficiency, central hypocortisolism, pituitary apoplexy, immune-mediated hypophysitis, diabetes insipidus, sick-euthyroid syndrome, subacute thyroiditis, and bone demineralization and enhanced fracture risk (13).

Finally, multisystem inflammatory syndrome in children (MIS-C), even relatively rare, poses a significant risk to children's health. This post-infectious condition is characterized by multisystem organ dysfunction that can be life-threating (14). With prompt management, most but not all children experience full recovery within weeks after initial presentation (15). However, long-term follow-up studies are limited. Thus, pediatric infectious disease specialists' role is of paramount importance for the coordination of the multidisciplinary approach that is essential for a favorable outcome of patients with MIS-C, but also for post-discharge follow-up.

In conclusion, the past experience on newly introduced infectious disease epidemics, the complexity of most of the post-COVID-19 sequelae and the current lack of in depth-knowledge about many of them are pragmatic arguments indicating that, at least initially, such sequelae require adult or pediatric infectious disease experts as coordinators of a multispecialty management which will lead to optimal HWRA (Figure 1). In later stages, and after stabilization of patient's health, the majority of these sequalae can be approachable by primary care itself.

**Figure 1:**
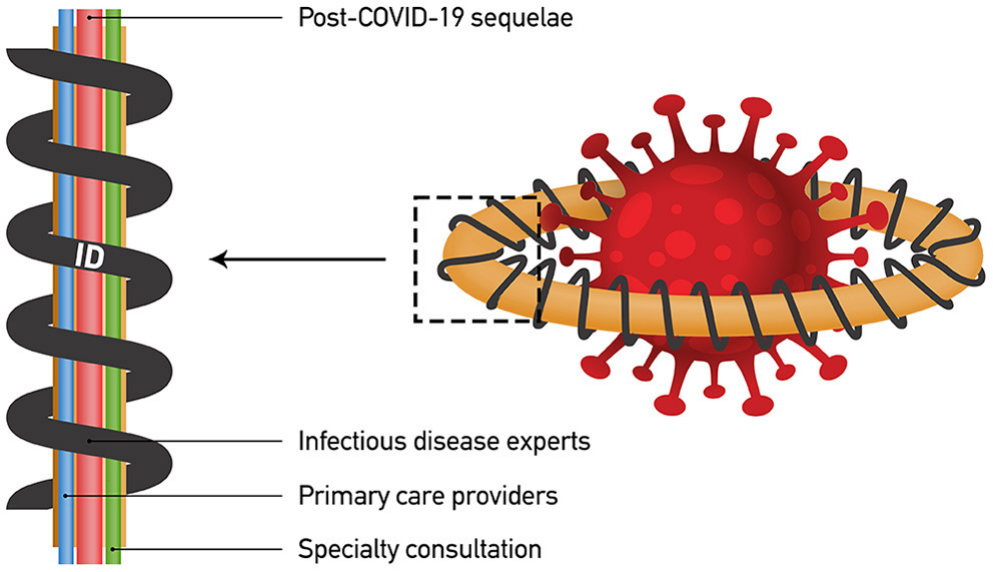
Multispecialty approach to confront post-COVID-19 sequalae.

## Author Contributions

All authors listed have made a substantial, direct, and intellectual contribution to the work and approved it for publication.

## Conflict of Interest

The authors declare that the research was conducted in the absence of any commercial or financial relationships that could be construed as a potential conflict of interest.

## Publisher's Note

All claims expressed in this article are solely those of the authors and do not necessarily represent those of their affiliated organizations, or those of the publisher, the editors and the reviewers. Any product that may be evaluated in this article, or claim that may be made by its manufacturer, is not guaranteed or endorsed by the publisher.
